# An Unusual Case of Fever in Paralytic Ileus

**DOI:** 10.7759/cureus.61671

**Published:** 2024-06-04

**Authors:** Shubhangi Kanitkar, Sai Priya Ande, Prasad Bagare, Akshata Borle, Muskaan Ahlawat

**Affiliations:** 1 Department of Medicine, Dr. D. Y. Patil Medical College, Hospital and Research Centre, Pune, IND

**Keywords:** rheumatology, joint pains, skin rash, fever of unknown origin, paralytic ileus, still’s disease

## Abstract

Still's disease is frequently a condition of exclusion for patients with an unidentified cause of fever. Accompanying symptoms typically include fever, arthralgia, and a transient skin rash. The underlying pathophysiology indicates an autoimmune origin. Diagnosis is primarily clinical, often utilizing the Yamaguchi criteria. The case in question involves a 19-year-old male presenting with high-grade fever and paralytic ileus. The patient received intravenous glucocorticoids and cyclophosphamide, resulting in a rapid clinical improvement. During the follow-up, tofacitinib was initiated based on the clinical response observed.

## Introduction

Still's disease is identified as an autoimmune disorder primarily associated with fever and joint pain, accompanied by an evanescent skin rash. It has been deemed to be a diagnosis of exclusion that is usually encountered as a pyrexia of unknown origin (PUO). Yamaguchi et al. were one of the first to develop a set of clinical classification criteria that have gained widespread usage due to their high sensitivity of 96.2% and specificity of 92.1% [1]. However, no reliable diagnostic criteria or biological markers exist that can predict the nature of the progression of the disease. The subsequent case study details a 19-year-old male who had a long history of fever and presented with paralytic ileus. He was suspected of having Still's disease after a response to treatment. Stress stemming from family, work, or health issues may potentially trigger this condition [2]. However, a similar case report has not been reported in the literature.

## Case presentation

**Figure 1:**
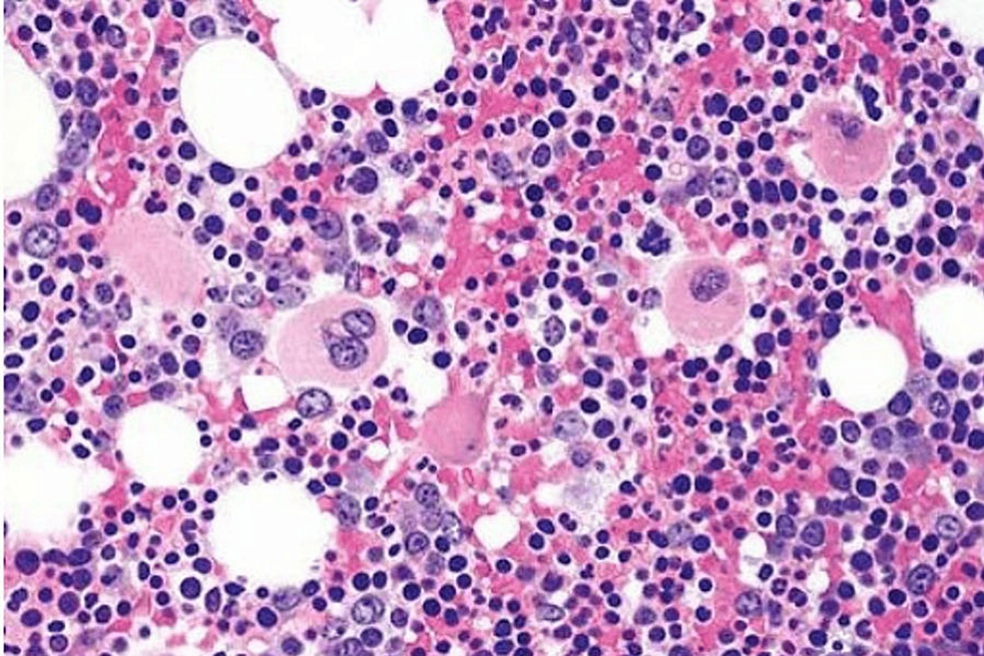
Bone marrow biopsy The figure depicts a normocellular marrow with active trilineage hematopoiesis.

**Figure 2:**
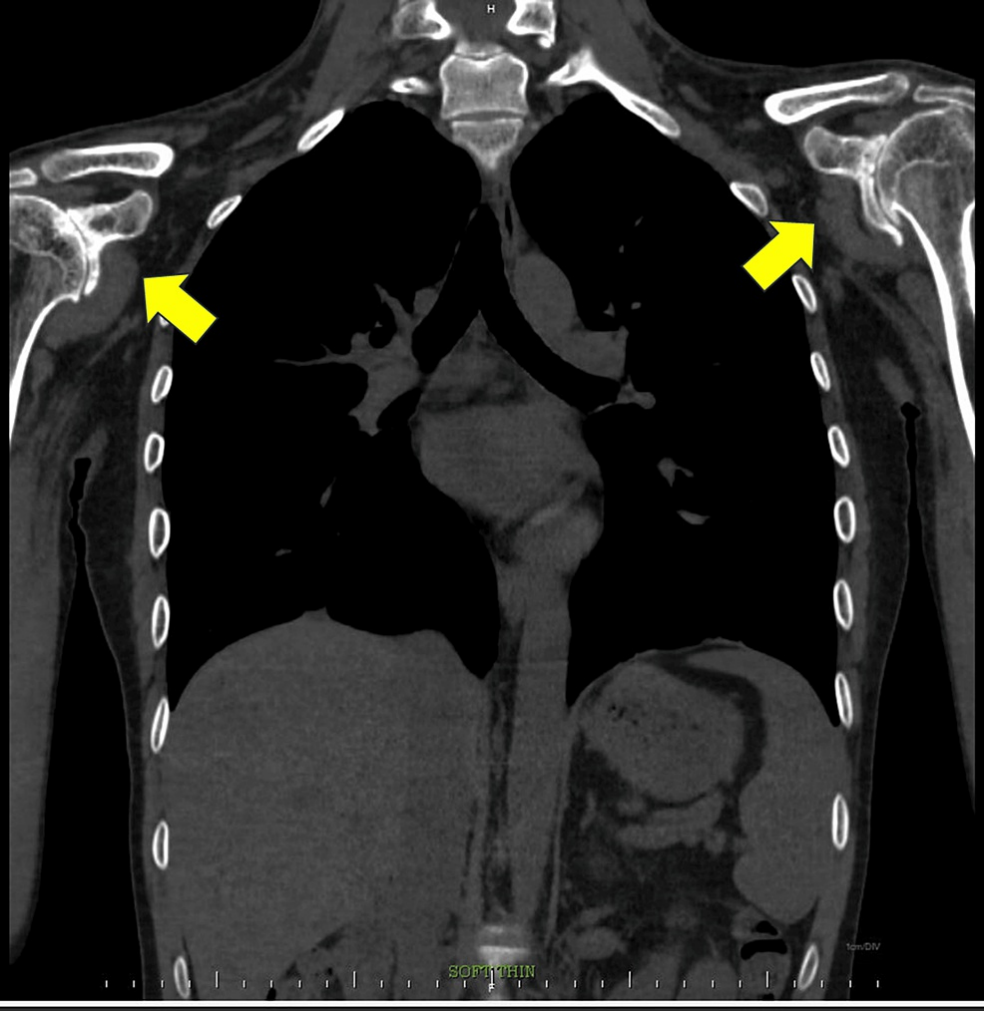
HRCT thorax: coronal oblique view No pleuro-parenchymal abnormalities were noted. Both the yellow arrows depict bilateral glenohumeral joints showing degenerative changes in the form of loss of humeral head contour, joint narrowing, marginal osteophytes, subchondral sclerosis, and marked osteopenia. Early changes of pseudoarthrosis were noted. HRCT thorax: high-resolution computed tomography of the thorax

**Figure 3:**
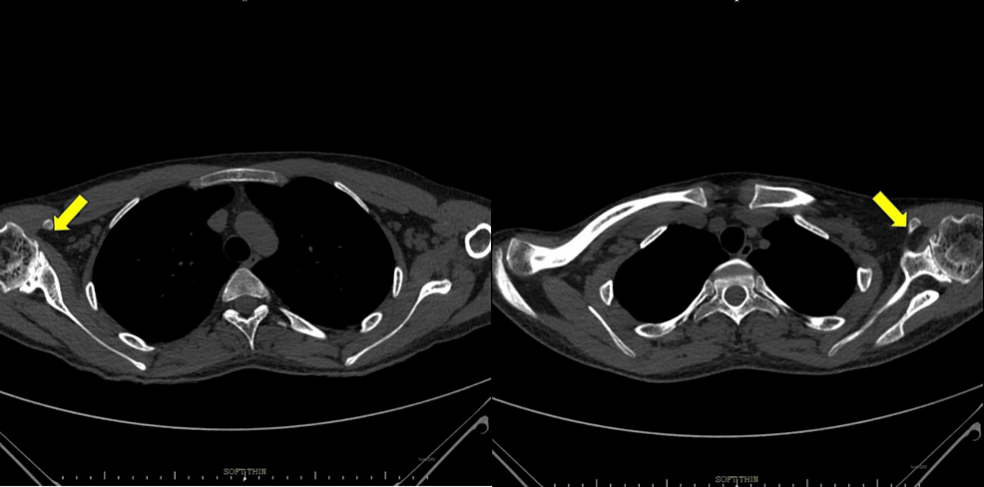
HRCT thorax: axial section No pleuro-parenchymal abnormality was noted. Both the yellow arrows depict bilateral glenohumeral joints showing degenerative changes in the form of loss of humeral head contour, joint narrowing, marginal osteophytes, subchondral sclerosis, and marked osteopenia. HRCT thorax: high-resolution computed tomography of the thorax

An adolescent male of 19 years presented with a two-month history of fever. He had four episodes of non-bilious vomiting and non-passage of flatus and feces for two days following the consumption of fast food. He was admitted to the surgical ward and was diagnosed with paralytic ileus secondary to hypokalemia (serum potassium 2.7 mmol/L on admission). He was managed conservatively with electrolyte and fluid correction, which resolved the abdominal complaints. During the course of hospital admission, the patient had a continuous high-grade fever of 101°F. He was tachycardiac with a heart rate of 105 beats per minute and normotensive with a systemic blood pressure of 110/80 mm Hg. After ruling out all foci of infection, he was considered a case of PUO. On probing further, the patient gave a history of inflammatory joint pains for seven years, primarily involving both knees, shoulders, and elbows. The comprehensive physical and systemic examinations yielded no notable abnormalities. Laboratory investigations indicated anemia, neutrophil-predominant leukocytosis, elevated liver transaminases, hyperferritinemia, an elevated erythrocyte sedimentation rate (ESR), and a high C-reactive protein (CRP) (Table 1). The bone marrow biopsy showed a normocellular bone marrow without any evidence of hemophagocytic lymphohistiocytosis (HLH) (Figure 1). Erosive arthritis was evident in the visible portions of the shoulder joint of high-resolution computed tomography of the thorax (HRCT thorax) (Figure 2, Figure 3). An echocardiogram suggested a mild, non-tappable pericardial effusion (Table 2). In view of the antecedent history of inflammatory joint pains, the patient was tested for anti-nuclear antibodies by immunofluorescence (ANA (IF)), anti-streptolysin O titre (ASO titre), and anti-cyclic citrullinated peptide (anti-CCP) that yielded negative results (Table 1). The clinical presentation, along with the fulfillment of more than five Yamaguchi criteria, has led to the diagnosis of adult-onset Still's disease (AOSD).

**Table 1:** Lab investigations Anti-CCP: anti-cyclic citrullinated peptide; ANA (IF): anti-nuclear antibody by immunofluorescence; ASO titre: anti-streptolysin O titre; gm%: gram percentage; mil/cu.mm: million/cubic millimeter; cu.mm: cubic millimeter; mg/dl: milligrams/deciliter; U/L: units/liter; IU/L: international units/liter; g/dl: grams/deciliter; mmol/L: millimoles/liter; mm/hr: millimeter/hour; ng/ml: nanograms/milliliter; HDL cholesterol: high-density lipoprotein cholesterol

Investigation	On admission	On follow-up	Reference values
Haemoglobin	7.3	10.8	14-16 gm%
Total leukocyte count	11.2	3.77	4.6-6.4 mil/cu.mm
Platelets	4,74,000	3,31,000	1,40,000-4,40,000/cu.mm
Total bilirubin	1.1	0.98	0.2-1.2 mg/dl
Aspartate transaminase	124	30.0	0-46 U/L
Alanine transaminase	267	18.6	0-49 U/L
Alkaline phosphatase	76	85	0-115 IU/L
Total proteins	6.6	6.2	6.2-8.1 g/dl
Albumin	2.6	3.2	3.6-5.5 g/dl
Globulin	4.0	3.0	3.5-5.0 g/dl
Urea	26	21	15-40 mg/dl
Creatinine	0.9	1.09	0.8-1.3 mg/dl
Sodium	131	130	136-145 mmol/L
Potassium	2.4	3.9	3.5-5.2 mmol/L
Chloride	101	104	95-106 mmol/L
Urine microscopy	Normal report		
Blood culture and sensitivity	No organism cultured		
Urine culture and sensitivity	No organism cultured		
Erythrocyte sedimentation rate	120	53	0-15 mm/hr
C-reactive protein	83	17	0.3-1.9 mg/dl
Ferritin	26,669	1200	12-300 ng/ml
Rheumatoid factor	Negative titre		
Anti-CCP	Negative titre		
ANA (IF)	Negative		
ANA blot	Negative		
ASO titre	Negative		
Widal test	1:80		>1:160
Total cholesterol	132 mg/dl		<200 mg/dl
Triglycerides	63 mg/dl		<150 mg/dl
HDL cholesterol	52 mg/dl		>40 mg/dl
Non-HDL cholesterol	70 mg/dl		

**Table 2:** Radiological investigations USG abdomen and pelvis: ultrasonography of the abdomen and pelvis; chest X-ray PA view: chest X-ray posterior-anterior view; HRCT thorax: high-resolution computed tomography of the thorax

Investigation	Report
USG abdomen and pelvis	Visualized bowel loops are not dilated and loaded with feces and show normal peristalsis. The spleen is normal in size and echotexture.
Chest X-ray PA view	Within normal limits
HRCT thorax	No obvious pleuro-parenchymal abnormality was noted. Changes of arthritis of the bilateral glenohumeral joints (right>left)
Echocardiogram	A thin rim of pericardial effusion was noted. Normal left ventricular ejection fraction: 60%

The patient received pulse glucocorticoid therapy with an injection of methylprednisolone (1 gram/kg) intravenously. Lab parameters showed a decreasing trend with clinical improvement. He was subsequently switched to intravenous cyclophosphamide. The patient was initially discharged with a prescription for oral steroids, which were gradually tapered off during follow-up visits. Eventually, the patient transitioned to disease-modifying antirheumatic drugs (DMARDs), i.e., Capsule tofacitinib 5 mg twice a day, for ongoing treatment.

## Discussion

AOSD is an inflammatory condition of autoimmune etiology that involves multiple systems. The foremost clinical case of Still's disease dates back to 1971 and was documented by Eric Bywaters, affecting a woman in her 30s with a decade-long history of periodic skin rashes and arthritis [3]. This disease engages both innate and adaptive immunity in its pathogenesis. The condition demonstrates a twin peak age distribution, primarily affecting individuals between 16 and 25 years and then those between 36 and 46 years. Although this pattern is common, it is not absolute, as cases have been reported in individuals older than 60 years as well [4]. Nirmala and colleagues suggest that systemic juvenile idiopathic arthritis (SJIA) affecting younger age groups and AOSD affecting adults may be varying phases of a single disease spectrum, as evidenced by the effectiveness of canakinumab in both age groups [5]. However, it has been observed that the clinical picture varies in children, adults, and the elderly [6,7].

The condition is deemed uncommon, with its prevalence estimated to range from 0.73 to 6.77 cases per 100,000 people [8-10]. While regional data is scarce, a prevalence rate of 3.9 per 100,000 individuals has been recorded in Japan, and a rate of 6.77 per 100,000 individuals has been noted in Turkey [8,10].

Fever is the ubiquitous presenting feature of Still's disease. The other manifestations include skin rash, arthritis, lymphadenopathy, myalgias, and hepatosplenomegaly [10]. A similar case report has not been reported in the literature. Elevated inflammatory markers, hyperferritinemia, leukocytosis, and ANA being negative were observed in the majority of cases that were diagnosed [4]. The most dreaded complication of Still's disease is macrophage activation syndrome (MAS), with a reported incidence of 26% [4]. The diagnosis of AOSD depends heavily on clinical presentation. Several diagnostic criteria exist for AOSD, but the Yamaguchi and the next in line, which are the Fautrel clinical classification criteria, are the most widely accepted [1,11] (Table 3). To support an AOSD diagnosis, the Yamaguchi criteria, in particular, require the presence of five criteria, two of which must be major.

**Table 3:** Yamaguchi criteria WBC: white blood cells; ANA: anti-nuclear antibody Reference: [1]

Major criteria	Minor criteria	Exclusion criteria
1. Fever more than equal to 39°C lasting for at least one week	1. Sore throat	Infections, malignancy, other autoinflammatory disorders (mainly polyarteritis nodosa and rheumatoid vasculitis with extra-articular features)
	2. Enlargement of the lymph nodes	
2. Neutrophil predominant leukocytosis with WBC >10,000/mm^3^; polymorphs >80%		
	3. Organomegaly-splenomegaly or hepatomegaly	
	4. Deranged liver function tests	
3. Arthralgia or arthritis lasting for more than two weeks		
	5. Negative ANA and rheumatoid factor tests	
4. Typical non-pruritic salmon pink skin rash		

The role of several biomarkers like interleukins (IL) (IL-37, IL-18, IL-6, and IL-1), glycosylated ferritin, heme oxygenase-1, and so on is being evaluated as prognostic markers and potential therapeutic targets [4]. Currently, there is no internationally recognized approach to the management of Still's disease. Several clinical practice guidelines have been proposed with different clinical management approaches. In 2018, a clinical practice guideline in Japan proposed the use of systemic glucocorticoids with pulse steroid therapy for the amelioration of severe systemic manifestations. The use of methotrexate was strongly recommended for steroid-refractory cases. Tocilizumab and canakinumab were proposed to have a steroid-sparing effect [12]. An expert group in Italy proposed the use of IL-1 inhibitors as the mainstay of treatment [13]. Feist et al. proposed an approach that advocates for the use of biologics early in the disease [14]. A general approach to early disease management involves the use of glucocorticoids, transitioning to synthetic DMARDs, and biologicals, depending on the patient's clinical response. In the described case, the patient exhibited a typical presentation of Still's disease with an unidentified cause of fever. However, the initial presentation of paralytic ileus has made it a challenging diagnosis. While the patient was thoroughly investigated and diagnosed, a delay in presentation needs to be pointed out. The unavailability of standard disease prognostication scores and markers points out the dilemma of future treatment courses.

## Conclusions

AOSD has garnered increased attention recently. Diagnosis primarily relies on clinical criteria and remains a diagnosis of exclusion. Significant unmet needs persist, notably the absence of reliable prognostic markers, comprehensive studies on treatment response, and understanding of clinical progression in AOSD. The case described above has had a delayed diagnosis owing to the delay in presentation to a hospital and also the mode of clinical presentation to the hospital. This underlines the multitude of presentations by AOSD. However, the response to treatment with timely follow-up is a satisfactory outcome of the diagnosis.
